# Residual Proviral Reservoirs: A High Risk for HIV Persistence and Driving Forces for Viral Rebound after Analytical Treatment Interruption

**DOI:** 10.3390/v13020335

**Published:** 2021-02-21

**Authors:** Xiaolei Wang, Huanbin Xu

**Affiliations:** Tulane National Primate Research Center, Division of Comparative Pathology, Tulane University School of Medicine, 18703 Three Rivers Road, Covington, LA 70433, USA; xwang@tulane.edu

**Keywords:** HIV, SIV, cell-associated viral reservoir, proviral reservoir

## Abstract

Antiretroviral therapy (ART) has dramatically suppressed human immunodeficiency virus (HIV) replication and become undetectable viremia. However, a small number of residual replication-competent HIV proviruses can still persist in a latent state even with lifelong ART, fueling viral rebound in HIV-infected patient subjects after treatment interruption. Therefore, the proviral reservoirs distributed in tissues in the body represent a major obstacle to a cure for HIV infection. Given unavailable HIV vaccine and a failure to eradicate HIV proviral reservoirs by current treatment, it is crucial to develop new therapeutic strategies to eliminate proviral reservoirs for ART-free HIV remission (functional cure), including a sterilizing cure (eradication of HIV reservoirs). This review highlights recent advances in the establishment and persistence of HIV proviral reservoirs, their detection, and potential eradication strategies.

## 1. Introduction

**Figure 1:**
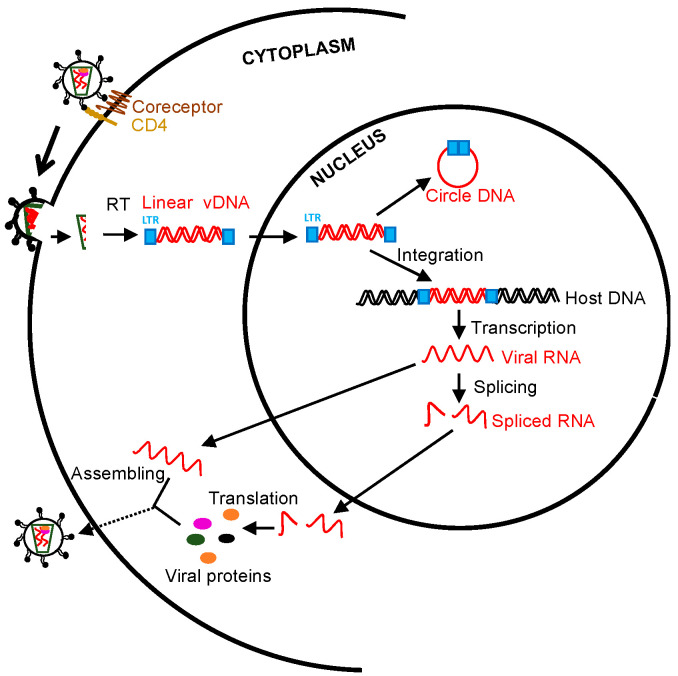
Schematic overview of human immunodeficiency virus (HIV)/ simian immunodeficiency virus (SIV) life cycle and measurable viral parameters. Viral particles enter target cells, followed by reverse transcription, integration, transcription, splicing, translation, and virion packaging. The unspliced viral RNAs are transcribed from the integrated provirus. The transported single 5′ capped genomic viral RNAs (~9 Kb) are assembled to the nascent virions. Two or three guanosines 5′ full-length viral RNAs directly translate viral proteins (gag and pol). Rev-dependent export of incompletely spliced RNAs (~4 Kb) to cytoplasm contributes to env, vif, vpr, and vpu expression, whereas rev-independent multiply spliced species (~2 Kb) constitutively express accessory and regulatory proteins (tat, rev, and nef). Cell-associated viral RNA transcripts and viral DNA can be directly measured [18,19,20].

In human immunodeficiency virus (HIV) life cycle, HIV RNA genome post-viral entry is reverse-transcribed into a double-stranded DNA, followed by transportation into the nucleus. Such a viral DNA/integrase complex preferentially integrates into the transcriptionally active sites of host chromosomes [1]. Notably, HIV DNA integration is an essential hallmark of viral production as integrated proviral DNA serves as a dominant template in the process of HIV replication, compared with unintegrated viral DNA forms [1,2,3]. By contrast, nonintegrated linear viral DNAs are unstable and accompanied by limited transcription to transiently express early viral elements [4,5,6,7], while nonintegrated long terminal repeat (LTR) circles, representing extrachromosomal bystander products that failed upon viral integration [8], basically lose the capacity of viral replication and are ultimately diluted due to cell proliferation [9]. However, the sporadic emergence of linear viral DNA and 2-LTR circles observed under ART is perhaps indicative of ongoing viral replication at low levels [6]. In the context of HIV replication from provirus transcription to virion assembling, unspliced RNA (~9-Kb) and more than 100 differentially spliced transcripts (predominant two-class sizes of early ~2-Kb and late ~4-Kb RNA) are generated [10,11]: the unspliced transcript is a template for gag/pol translation and viral RNA genome packaging; 4Kb incompletely spliced transcripts encode viral proteins env, vif, vpr, and vpu following export into the cytoplasm; and multiply spliced viral RNAs (~2 Kb) express regulatory tat, rev, and nef for transactivation and nuclear export of the viral RNAs [12,13,14,15,16,17,18,19,20] (Figure 1). Therefore, various cell-associated HIV RNA/DNA forms may reflect the different viral replication status and clinical significance [8,21,22,23,24,25,26]. For example, HIV gag RNA transcripts represent bona fide genomic HIV RNA or gag and gag-pol polyproteins in viral replication [27,28]; spliced HIV tat/rev RNAs are functional for viral replication and production [29]; and integrated proviral DNA is a marker to estimate the proviral reservoirs [22,24,30,31,32,33,34]. A small number of HIV-infected cells harboring proviral DNA may replenish proviral reservoirs through clonal expansion or cell division, maintaining viral persistence [9,35,36,37,38,39].

## 2. Establishment and Anatomical Distribution of Proviral Reservoirs

**Figure 2:**
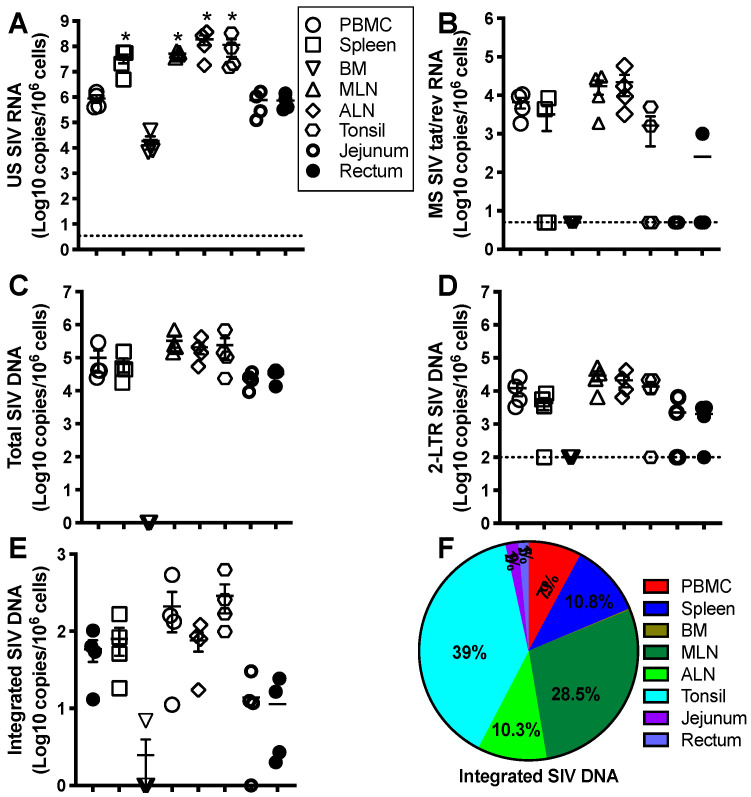
Representative levels and anatomical tissue distribution of cell-associated SIV RNA/DNA in SIV-infected macaques. Adult Indian-origin rhesus macaques (Macaca mulatta) were intravenously inoculated with 100 TCID50 SIVmac251. After 8 weeks, these animals received three anti-HIV drugs (TFV 20 mg/kg/day; FTC 30 mg/kg/day and DTG 2.5 mg/kg/day) for 20 months. The levels of cell-associated unspliced (US) SIV RNA (**A**), multiply spliced (MS) SIV tat/rev RNA (**B**), total SIV DNA (**C**), circular SIV 2-long terminal repeat (LTR) (**D**), and integrated proviral DNA (**E**), in blood, spleen, mesenteric lymph node, axillary lymph node, jejunum, and rectum from SIV-infected animals 3 months after ATI, reaching the levels prior to treatment. (**F**) Distribution of proviral reservoir in tissues examined. Note that integrated proviral DNA was predominantly distributed in peripheral blood and lymphoid compartments, and rapidly increased to pre-treatment levels after ATI. Cell-associated SIV RNA/DNA are expressed as copies per one-million cells. * *p* < 0.01, compared with PBMCs.

Given numerous, substantial differences in the immunology of humans and mice [40], nonhuman primate models (NHPs) provide an excellent model of human immunology, diseases, and therapies due to their remarkably similar genomes, physiology, and immune systems [41,42,43]. In addition, it is challenging to access sufficient tissues for analysis in humans because of sample collection limitations. It is especially difficult to evaluate the proviral reservoir seeding that may lead to viral dormancy, “occult” infection, or viral rebound after treatment interruption [44]. Simian immunodeficiency viruses (SIVs) and chimeric simian–human immunodeficiency viruses (SHIVs) that carry HIV envelope from transmitted founder (T/F) viruses are widely used in nonhuman primate models to recapitulate HIV infection in humans, including HIV transmission, pathogenesis, viral latency, and curative strategies [45,46,47,48,49]. Early six-month ART is initiated at 6h, or day 1, 2, and 3 post-SIV infection in rhesus macaques. Once treatment is discontinued, counterpart viral rebound is respectively shown at 0%, 20%, 60%, and 100% of animals, which correlates with levels of integrated viral DNA in LN CD4+ T cells [50], suggesting that the proviral reservoirs are rapidly seeded post-HIV/SIV infection, resistant to ART. In lymphoid tissues that represent typical sanctuary sites for HIV, anti-HIV drugs maintain suboptimal levels in the follicle and germinal center niches [51,52,53,54], and virus-infected cells are shielded from specific CD8+ T cell responses [55]. All of these may maintain residual proviral reservoirs in lymphoid tissues in HIV+ subjects, even on lifelong ART. The size of the HIV-1 reservoir differs in tissues, with the frequency of infection generally higher on a per-cell basis in the tonsils, lymph nodes (LN), gut-associated lymphoid tissues (GALT), and spleen, which basically contain abundant organized lymphoid structures [56,57,58]. Our recent study shows that the levels of cell-associated SIV RNA/DNA in PBMCs and lymph nodes increase in rhesus macaques on prolonged ART (>20 months) after ATI [59]. Further examination of anatomical distribution and size of the viral reservoir (multiple cell-associated viral parameters) after ATI indicated that all cell-associated RNA/DNAs were recovered to the levels prior to treatment in various tissues (blood, spleen, axillary and mesenteric lymph node, jejunum, and rectum), except bone marrow, in which viral nucleic acid was undetectable, yet jejunum and rectum had very low levels of MS SIV RNA, probably attributing to massive depletion of CD4+ T cells in GALTs. Lymphoid tissues maintained higher US SIV transcript levels and stable MS SIV RNA (Figure 2A,B), while levels of total SIV DNA and 2-LTR DNA were equivalent in all tissues examined (Figure 2C,D). Most notably, integrated proviral DNA was detected in all tissues (detectable in bone marrow from only 1 of 4 animals), yet also higher in peripheral and lymphoid tissues (Figure 2E,F), consistent with converging evidence that systemic and lymphoid tissue compartments may serve as important sites for viral reservoirs [54,60,61,62,63]. These findings also support the conception that viral reservoirs persist in multiple tissues, highlighting the rapid recovery and replenishment of HIV reservoirs in tissues once anti-HIV treatment is discontinued.

CD4+ T cells are primary target cells of HIV/SIV, constituting the predominant productive and latent reservoirs [36,64]. CD4+ T cells harboring proviral DNA may mostly contribute to persistent HIV/SIV infection [65]. The HIV latently infected cells are resting memory CD4+ T cells with regenerative potential, including T memory stem cells (Tscm) and central memory cells (Tcm) [36,64], and also include macrophages and dendritic cells in various tissues [66,67,68,69]. Our previous studies indicate that organized lymphoid tissues are major sites for persistent SIV reservoirs, in which follicular T helper (Tfh) cells are differentiated and accumulated in chronic stage and contain high levels of both SIV RNA and proviral DNA [53,70]. Of these, elevated pro-inflammatory cytokines and continuous antigen persistence may drive Tfh precursor differentiation toward Tfh cells, resulting in abnormal accumulation of productively and latently infected Tfh cells [53,71,72,73]. In primary infection, we also identify that Th17 cells are the initial foci of SIV infection in vaginal tissues [74]. In addition to CD4+ T cells, recent advances reveal that the tissue-resident myeloid cells (e.g., monocytes and macrophages) in the brain and other tissues might be a resource of HIV reservoir throughout ART [75,76,77,78,79]. However, myeloid cells in the blood and colon likely contain HIV transcripts, but few proviruses are detected in a large fraction of HIV+ patients under ART, compared with CD4+ T cells with readily detectable proviral DNA [80,81]. In terms of myeloid cells, circulating monocytes might not be considered reservoirs due to their infrequent HIV infection, low levels of proviral DNA, and short life span [81], while macrophages are probably another long-lived cellular reservoir for viral persistence, viral rebound, and reestablishment of productive HIV infection when treatment is interrupted [82,83,84,85,86,87,88].

The markers of HIV reservoirs are desperately required to determine whether ART should resume [89,90]. Early indicators that can predict resurgence of viremia after ATI may aid treatment decisions in people living with HIV. Ideally, a predictor of viral rebound would be measured prior to ART interruption to gauge whether an HIV-infected individual could safely stop ART. It is reported that T-cell exhaustion markers, including PD–1, Tim–3, and Lag–3, strongly predict time to the return of viraemia [91], yet this is questionable because functional T-cell response is not the only determinant in the viral containment of aviremic patients [92]. A number of markers for productively or latently HIV-infected cells with enrichment of HIV DNA, such as CD2 [93], CD20 [94], CD30 [95], and CD32a [96], are still controversial and require further confirmation [97,98,99,100]. Notably, it is the small HIV-integrated proviral reservoirs that are distributed in various lymphocyte subsets of anatomical sites for persistence of replication-competent viruses. These reservoirs become a driving force for viral rebound after ATI. However, markers of proviral reservoirs are multifactorial and complex. For example, heterogeneous proviral reservoirs exist in various anatomic tissues but not merely in blood, and their measurement also lies in ultrasensitive assay. The proviral reservoirs also laboriously analyze the intact viral genome from tissues in the body of patients and perform limited proviral assessment by integration site analysis [101,102]. Despite viral DNA levels being a potential cause of viral rebound, some patients with low-viral DNA levels do not show delayed viremia rebound [22,103]. Our recent study indicating an increased ratio of proviral DNA after ATI likely better predicts the emergence and degree of viral rebound [59], yet more reliable markers are still needed toward developing a cure for HIV.

## 3. Impact of Early Antiretroviral Therapy on the Proviral Reservoir Elimination and Sustained Virologic Remission

Early ART, even initiated as early as one day of infection in adult subjects or within 30 min after birth in infants, usually fails to achieve a sustained state of ART-free virologic remission, leading to HIV rebound after months or years of treatment interruption [50,104,105,106,107,108,109,110,111,112,113]. Although the Berlin, London, and Düsseldorf adult patients seemingly appear to have a “sterilizing HIV cure” [108,114,115,116,117], the treatment of these three cases, which involved toxic chemotherapy due to hematological malignancies followed by hematopoietic stem cell transplantation, is not feasible in others because of its complexity and risk. Rapid establishment of proviral reservoir harboring replication-competent virus remains the major obstacle to HIV cure or remission. Early ART in adults infected with HIV, even if initiated within days of infection, has no curative effects [104,105,106]. Additionally, ART initiation in adult animals, from 4 to 14 dpi, fails to prevent the virus from spreading and reservoir seeding, leading to viral rebound within four months ATI [118,119,120]. Distinct from those in adults, the immune system in newborn infants is compartmentalized by the “immature” systemic immune system and the “functional/mature” mucosal system [121,122]. Thus, viral susceptibility, viral reservoir seeding, and immune responses in developing neonates exposed to HIV might differ from those in adults [123,124]. Immediate initiation of ART, ideally within hours after birth, may restrict viral reservoir size, maintain normal neonatal immune development, and possibly provide opportunities for being drug-free after going off ART [44,110,125,126,127,128,129,130]. However, in the pediatric AIDS clinical trials and cases, there is no precedent to achieve HIV remission in the HIV+ infants treated by early ART; eventual viral rebound is observed once treatment is discontinued [107,108,124,126,130,131,132,133,134,135]. Conceivably, integrated proviral reservoirs established in anatomic tissues cannot be completely eliminated by ART [22,129,136,137,138], explaining why they contribute to viral rebound after ATI. Together, these findings highlight that a small number of integrated proviral reservoirs seeded could be a key hurdle to advance HIV treatment towards a cure.

## 4. Measurement of HIV Reservoirs

As described in HIV life cycles, the existence and abundance of HIV RNA/DNA represent different infectious status and clinical significance [8,21,22,23,24,25,26]. The proviral reservoir could be a potential indicator to produce infectious progeny for the viral rebound after ATI. The advantages and disadvantages of the HIV reservoir measurements have been recently reviewed and discussed [139]. Current scalable assays to measure viable proviral reservoirs may underestimate the bona fide replication-competent reservoir [31,140,141,142,143,144,145,146,147], including Quantitative Viral Outgrowth Assays (QVOA) that assess the size of the replication-competent HIV latency in resting CD4+ T cells under conditions with latency reactivation [26,144,148,149], and Tat/rev-Induced limiting Dilution Assays (TILDA) that measure the frequency of productively HIV-infected cells with inducible multiply-spliced HIV transcripts [140,142,150], in which a proportion of intact proviral DNA may be non-reactivable in the host for a lifetime [144,151,152]. They may even maintaining repressive silence when intact proviral DNA integrates into the “gene desert” sites of chromosomes enriched in repressive chromatin marks [153]. In comparison, quantitative PCR [31,140,141,142,143,144,145,146] and near-Full-Length Individual Proviral sequencing (FLIPS) [154] may overestimate the proviral reservoir. More than 90% of proviruses in ART-treated patients might be replicated defective because of internal deletions, mutations, premature stop codons, or defects in splicing and packaging signals [146,155,156,157]. However, these defective proviruses may partially express viral proteins, eliciting host responses [145,158,159]. Proviral “quasispecies” may be distributed on distinct chromosomal sites in single cells of various tissues [160], and intact proviruses may not necessarily produce replication-competent virions at both transcription and translation levels [146,161,162,163], weakening the interpretation of the results from assays above. Although sensitive and practical assays are well developed, there is generally no single “gold-standard” approach to reliably evaluate proviral reservoirs in systemic and lymphoid tissues [140,150,163,164]. Biomarkers are still desperately needed to assess viral persistence, effective therapy, and treatment resumption.

## 5. Strategies to Eradicate Viral Reservoirs

The persistence of latent HIV-infected cellular proviral reservoirs represents the major hurdle to virus eradication in patients treated with ART. Since the transcription of HIV genes depends on cell activation state, integrated HIV DNA is transcriptionally silent in these cells and therefore unaffected by ART [165]. Therefore, various cure strategies are proposed toward HIV cure (Table 1) [114,116,117,166,167,168,169,170,171,172,173,174,175]. Of these, “shock and kill” and “block and lock” strategies are attempted to reactivate HIV-1 latency or to create a deep latent state. In “shock and kill”, combined with ART, cells harboring latent HIV provirus are activated by cytokines (e.g., IL-2), lipopolysaccharides, bacterial superantigens, anti-T cell antibodies (OKT3), histone deacetylase inhibitors/HDACi (SAHA), or protein kinase agonists. Once activated, these cells could be eliminated through viral cytopathic effects or host cytolytic T lymphocytes (CTL) responses [176,177]. However, most, if not all of these agents, are not effective in fully reactivating HIV latency in cells from patients on ART or reducing the size of latent reservoirs [178,179,180,181,182]. It remains uncertain whether ART combined with immune activation strategies could eventually (or ever) eliminate all productively infected cells through viral cytopathic effects or other immune mechanisms [166,179,183,184,185]. In the alternative “block and lock” approach, latency-promoting agents are applied to permanently prevent latency reactivation and replenishment [172,186]. However, this approach essentially lacks specificity of cells containing residual viral genome and efficiency in deep tissues, likely leading to lifelong treatment-induced adverse outcomes and complications. Given that extracellular HIV envelope glycoprotein (Env) could be presented on the productively HIV-infected cells, these cells could be recognized by anti-HIV antibody drug (e.g., toxin or radionuclides) conjugates [187,188] or broadly neutralizing antibodies (bnAbs) [173,189,190], likely leading to specifically and selectively killing of residual HIV-infected cells. However, current passive therapy by bnAbs does not fully eradicate proviral reservoir with potential in selecting escape variants and eventual viral rebound in ART-treated subjects after treatment cessation [191,192,193,194,195]. Meanwhile, although novel genome-editing technology is promising to disrupt or ablate proviral genome, it requires more advances in vivo delivery and specificity of target cells [196,197,198].

## 6. Perspective

HIV cure remains the greatest challenge for therapeutic strategies. The existence of small cellular reservoirs containing an integrated intact viral genome is a major obstacle in finding a cure for HIV infection. Lifelong treatment by antiretroviral drugs or latency-reversing or promoting agents predisposes one to a high risk of physiological function and complications. The other challenges may also lie in discrepant latent status in individual HIV+ subjects and full evaluation of the proviral reservoir, not only in peripheral blood, owing to limitations in human sample collection. Among cure strategies, novel gene-editing applications in vivo might be able to potentially ablate HIV genome from cellular proviral reservoirs, albeit far more studies are still needed.

## Figures and Tables

**Table 1:** General HIV curative strategies.

HIV Cure Strategy	Goal	Efficacy	Limitations
**ART**	HIV cure	Suppression of HIV replication	Failure to cure HIV
**Vaccine**	Prophylactic and therapeutic effects against HIV infection	Protective immune responses	No successful vaccine to date
**Shock and Kill**	HIV latency reactivation, cytolytic effects of target cells	Partial latency reactivation	Failure or limited reservoir perturbation
**Lock and block**	Permanent silencing of HIV latency	Suppression of HIV reactivation	Delivery difficulty and safety
**bnAb therapy**	Long-term sustained HIV remission	Suppression of sensitive HIV strain	Emergence of resistant viruses, likely limited effect on viral reservoirs
**Gene editing**	Disruption of viral genome or HIV coreceptors	Ablation of viral genome	Difficulties of delivery and specificity in vivo, antigenicity, and genotoxicity
**Stem cell transplantation**	Immune reconstitution and CCR5 mutation	Cases of “sterilizing HIV cure”	Combinations of toxic chemotherapy with transplantation are not feasible in others

## Data Availability

All data generated during this study are included in this article.

## References

[1] Craigie R., Bushman F.D. (2012). HIV DNA integration. Cold Spring Harb. Perspect. Med..

[2] Schroder A.R., Shinn P., Chen H., Berry C., Ecker J.R., Bushman F. (2002). HIV-1 integration in the human genome favors active genes and local hotspots. Cell.

[3] Maldarelli F. (2016). The role of HIV integration in viral persistence: No more whistling past the proviral graveyard. J. Clin. Investig..

[4] Koelsch K.K., Liu L., Haubrich R., May S., Havlir D., Gunthard H.F., Ignacio C.C., Campos-Soto P., Little S.J., Shafer R. (2008). Dynamics of total, linear nonintegrated, and integrated HIV-1 DNA in vivo and in vitro. J. Infect. Dis..

[5] Wu Y., Marsh J.W. (2003). Early transcription from nonintegrated DNA in human immunodeficiency virus infection. J. Virol..

[6] Sloan R.D., Wainberg M.A. (2011). The role of unintegrated DNA in HIV infection. Retrovirology.

[7] Hamid F.B., Kim J., Shin C.G. (2017). Distribution and fate of HIV-1 unintegrated DNA species: A comprehensive update. AIDS Res. Ther..

[8] Policicchio B.B., Cardozo E.F., Sette P., Xu C., Haret-Richter G., Dunsmore T., Apetrei C., Pandrea I., Ribeiro R.M. (2018). Dynamics of Simian Immunodeficiency Virus Two-Long-Terminal-Repeat Circles in the Presence and Absence of CD8^+^ Cells. J. Virol..

[9] Anderson E.M., Maldarelli F. (2018). The role of integration and clonal expansion in HIV infection: Live long and prosper. Retrovirology.

[10] Ocwieja K.E., Sherrill-Mix S., Mukherjee R., Custers-Allen R., David P., Brown M., Wang S., Link D.R., Olson J., Travers K. (2012). Dynamic regulation of HIV-1 mRNA populations analyzed by single-molecule enrichment and long-read sequencing. Nucleic Acids Res..

[11] Purcell D.F., Martin M.A. (1993). Alternative splicing of human immunodeficiency virus type 1 mRNA modulates viral protein expression, replication, and infectivity. J. Virol..

[12] Karn J., Stoltzfus C.M. (2012). Transcriptional and posttranscriptional regulation of HIV-1 gene expression. Cold Spring Harb. Perspect. Med..

[13] Jayaraman B., Crosby D.C., Homer C., Ribeiro I., Mavor D., Frankel A.D. (2014). RNA-directed remodeling of the HIV-1 protein Rev orchestrates assembly of the Rev-Rev response element complex. eLife.

[14] Williamson J.R. (2015). Really exasperating viral protein from HIV. eLife.

[15] Spector C., Mele A.R., Wigdahl B., Nonnemacher M.R. (2019). Genetic variation and function of the HIV-1 Tat protein. Med. Microbiol. Immunol..

[16] Van der Velden G.J., Klaver B., Das A.T., Berkhout B. (2012). Upstream AUG codons in the simian immunodeficiency virus SIVmac239 genome regulate Rev and Env protein translation. J. Virol..

[17] Erkelenz S., Poschmann G., Theiss S., Stefanski A., Hillebrand F., Otte M., Stuhler K., Schaal H. (2013). Tra2-mediated recognition of HIV-1 5’ splice site D3 as a key factor in the processing of vpr mRNA. J. Virol..

[18] Pasternak A.O., Lukashov V.V., Berkhout B. (2013). Cell-associated HIV RNA: A dynamic biomarker of viral persistence. Retrovirology.

[19] Kuzembayeva M., Dilley K., Sardo L., Hu W.S. (2014). Life of psi: How full-length HIV-1 RNAs become packaged genomes in the viral particles. Virology.

[20] Dubois N., Marquet R., Paillart J.C., Bernacchi S. (2018). Retroviral RNA Dimerization: From Structure to Functions. Front. Microbiol..

[21] Fischer M., Joos B., Hirschel B., Bleiber G., Weber R., Gunthard H.F. (2004). Swiss HIV Cohort Study. Cellular viral rebound after cessation of potent antiretroviral therapy predicted by levels of multiply spliced HIV-1 RNA encoding nef. J. Infect. Dis..

[22] Williams J.P., Hurst J., Stohr W., Robinson N., Brown H., Fisher M., Kinloch S., Cooper D., Schechter M., Tambussi G. (2014). HIV-1 DNA predicts disease progression and post-treatment virological control. eLife.

[23] Hong F., Aga E., Cillo A.R., Yates A.L., Besson G., Fyne E., Koontz D.L., Jennings C., Zheng L., Mellors J.W. (2016). Novel Assays for Measurement of Total Cell-Associated HIV-1 DNA and RNA. J. Clin. Microbiol..

[24] Kiselinova M., De Spiegelaere W., Buzon M.J., Malatinkova E., Lichterfeld M., Vandekerckhove L. (2016). Integrated and Total HIV-1 DNA Predict Ex Vivo Viral Outgrowth. PLoS Pathog..

[25] Pasternak A.O., Berkhout B. (2018). What do we measure when we measure cell-associated HIV RNA. Retrovirology.

[26] Lu C.L., Pai J.A., Nogueira L., Mendoza P., Gruell H., Oliveira T.Y., Barton J., Lorenzi J.C.C., Cohen Y.Z., Cohn L.B. (2018). Relationship between intact HIV-1 proviruses in circulating CD4^+^ T cells and rebound viruses emerging during treatment interruption. Proc. Natl. Acad. Sci. USA.

[27] Pasternak A.O., DeMaster L.K., Kootstra N.A., Reiss P., O’Doherty U., Berkhout B. (2016). Minor Contribution of Chimeric Host-HIV Readthrough Transcripts to the Level of HIV Cell-Associated gag RNA. J. Virol..

[28] Baxter A.E., Niessl J., Fromentin R., Richard J., Porichis F., Charlebois R., Massanella M., Brassard N., Alsahafi N., Delgado G.G. (2016). Single-Cell Characterization of Viral Translation-Competent Reservoirs in HIV-Infected Individuals. Cell Host Microbe.

[29] Procopio F.A., Fromentin R., Kulpa D.A., Brehm J.H., Bebin A.G., Strain M.C., Richman D.D., O’Doherty U., Palmer S., Hecht F.M. (2015). A Novel Assay to Measure the Magnitude of the Inducible Viral Reservoir in HIV-infected Individuals. EBioMedicine.

[30] Yerly S., Gunthard H.F., Fagard C., Joos B., Perneger T.V., Hirschel B., Perrin L., Swiss HIV Cohort Study (2004). Proviral HIV-DNA predicts viral rebound and viral setpoint after structured treatment interruptions. AIDS.

[31] Avettand-Fenoel V., Hocqueloux L., Ghosn J., Cheret A., Frange P., Melard A., Viard J.P., Rouzioux C. (2016). Total HIV-1 DNA, a Marker of Viral Reservoir Dynamics with Clinical Implications. Clin. Microbiol. Rev..

[32] Rouzioux C., Avettand-Fenoel V. (2018). Total HIV DNA: A global marker of HIV persistence. Retrovirology.

[33] Cillo A.R., Hong F., Tsai A., Irrinki A., Kaur J., Sloan D.D., Follen M., Geleziunas R., Cihlar T., Win S.S. (2018). Blood biomarkers of expressed and inducible HIV-1. AIDS.

[34] Bachmann N., von Siebenthal C., Vongrad V., Turk T., Neumann K., Beerenwinkel N., Bogojeska J., Fellay J., Roth V., Swiss HIV Cohort Study (2019). Determinants of HIV-1 reservoir size and long-term dynamics during suppressive ART. Nat. Commun..

[35] Maldarelli F., Wu X., Su L., Simonetti F.R., Shao W., Hill S., Spindler J., Ferris A.L., Mellors J.W., Kearney M.F. (2014). HIV latency. Specific HIV integration sites are linked to clonal expansion and persistence of infected cells. Science.

[36] Chomont N., El-Far M., Ancuta P., Trautmann L., Procopio F.A., Yassine-Diab B., Boucher G., Boulassel M.R., Ghattas G., Brenchley J.M. (2009). HIV reservoir size and persistence are driven by T cell survival and homeostatic proliferation. Nat. Med..

[37] Maldarelli F. (2015). HIV-infected cells are frequently clonally expanded after prolonged antiretroviral therapy: Implications for HIV persistence. J. Virus Erad..

[38] Kwon K.J., Siliciano R.F. (2017). HIV persistence: Clonal expansion of cells in the latent reservoir. J. Clin. Investig..

[39] Ferris A.L., Wells D.W., Guo S., Del Prete G.Q., Swanstrom A.E., Coffin J.M., Wu X., Lifson J.D., Hughes S.H. (2019). Clonal expansion of SIV-infected cells in macaques on antiretroviral therapy is similar to that of HIV-infected cells in humans. PLoS Pathog..

[40] Mestas J., Hughes C.C. (2004). Of mice and not men: Differences between mouse and human immunology. J. Immunol..

[41] Hirsch V.M., Lifson J.D. (2000). Simian immunodeficiency virus infection of monkeys as a model system for the study of AIDS pathogenesis, treatment, and prevention. Adv. Pharmacol..

[42] Staprans S.I., Feinberg M.B., Shiver J.W., Casimiro D.R. (2010). Role of nonhuman primates in the evaluation of candidate AIDS vaccines: An industry perspective. Curr. Opin. HIV AIDS.

[43] Deere J.D., Schinazi R.F., North T.W. (2011). Simian immunodeficiency virus macaque models of HIV latency. Curr. Opin. HIV AIDS.

[44] Ananworanich J., Puthanakit T., Suntarattiwong P., Chokephaibulkit K., Kerr S.J., Fromentin R., Bakeman W., Intasan J., Mahanontharit A., Sirivichayakul S. (2014). Reduced markers of HIV persistence and restricted HIV-specific immune responses after early antiretroviral therapy in children. AIDS.

[45] Del Prete G.Q., Lifson J.D., Keele B.F. (2016). Nonhuman primate models for the evaluation of HIV-1 preventive vaccine strategies: Model parameter considerations and consequences. Curr. Opin. HIV AIDS.

[46] Parsons M.S., Le Grand R., Kent S.J. (2018). Neutralizing Antibody-Based Prevention of Cell-Associated HIV-1 Infection. Viruses.

[47] Chen Z. (2018). Monkey Models and HIV Vaccine Research. Adv. Exp. Med. Biol..

[48] Naranjo-Gomez M., Pelegrin M. (2019). Vaccinal effect of HIV-1 antibody therapy. Curr. Opin. HIV AIDS.

[49] Horsburgh B.A., Palmer S. (2019). For Viral Reservoir Studies, Timing Matters. Trends Microbiol..

[50] Whitney J.B., Lim S.Y., Osuna C.E., Kublin J.L., Chen E., Yoon G., Liu P.T., Abbink P., Borducci E.N., Hill A. (2018). Prevention of SIVmac251 reservoir seeding in rhesus monkeys by early antiretroviral therapy. Nat. Commun..

[51] Fletcher C.V., Staskus K., Wietgrefe S.W., Rothenberger M., Reilly C., Chipman J.G., Beilman G.J., Khoruts A., Thorkelson A., Schmidt T.E. (2014). Persistent HIV-1 replication is associated with lower antiretroviral drug concentrations in lymphatic tissues. Proc. Natl. Acad. Sci. USA.

[52] Fukazawa Y., Lum R., Okoye A.A., Park H., Matsuda K., Bae J.Y., Hagen S.I., Shoemaker R., Deleage C., Lucero C. (2015). B cell follicle sanctuary permits persistent productive simian immunodeficiency virus infection in elite controllers. Nat. Med..

[53] Xu H., Wang X., Malam N., Aye P.P., Alvarez X., Lackner A.A., Veazey R.S. (2015). Persistent Simian Immunodeficiency Virus Infection Drives Differentiation, Aberrant Accumulation, and Latent Infection of Germinal Center Follicular T Helper Cells. J. Virol..

[54] Wong J.K., Yukl S.A. (2016). Tissue reservoirs of HIV. Curr. Opin. HIV AIDS.

[55] Collins D.R., Gaiha G.D., Walker B.D. (2020). CD8^+^ T cells in HIV control, cure and prevention. Nat. Rev. Immunol..

[56] Yukl S.A., Shergill A.K., Ho T., Killian M., Girling V., Epling L., Li P., Wong L.K., Crouch P., Deeks S.G. (2013). The distribution of HIV DNA and RNA in cell subsets differs in gut and blood of HIV-positive patients on ART: Implications for viral persistence. J. Infect. Dis..

[57] North T.W., Higgins J., Deere J.D., Hayes T.L., Villalobos A., Adamson L., Shacklett B.L., Schinazi R.F., Luciw P.A. (2010). Viral sanctuaries during highly active antiretroviral therapy in a nonhuman primate model for AIDS. J. Virol..

[58] Sigal A., Kim J.T., Balazs A.B., Dekel E., Mayo A., Milo R., Baltimore D. (2011). Cell-to-cell spread of HIV permits ongoing replication despite antiretroviral therapy. Nature.

[59] Ziani W., Shao J., Wang X., Russell-Lodrigue K., Liu Y.Z., Montaner L.J., Veazey R.S., Xu H. (2021). Increased proviral DNA in circulating cells correlates with plasma viral rebound in SIV-infected rhesus macaques after antiretroviral therapy interruption. J. Virol..

[60] Couturier J., Lewis D.E. (2018). HIV Persistence in Adipose Tissue Reservoirs. Curr. HIV/AIDS Rep..

[61] Denton P.W., Sogaard O.S., Tolstrup M. (2019). Impacts of HIV Cure Interventions on Viral Reservoirs in Tissues. Front. Microbiol..

[62] Estes J.D., Kityo C., Ssali F., Swainson L., Makamdop K.N., Del Prete G.Q., Deeks S.G., Luciw P.A., Chipman J.G., Beilman G.J. (2017). Defining total-body AIDS-virus burden with implications for curative strategies. Nat. Med..

[63] Chaillon A., Gianella S., Dellicour S., Rawlings S.A., Schlub T.E., De Oliveira M.F., Ignacio C., Porrachia M., Vrancken B., Smith D.M. (2020). HIV persists throughout deep tissues with repopulation from multiple anatomical sources. J. Clin. Investig..

[64] Buzon M.J., Sun H., Li C., Shaw A., Seiss K., Ouyang Z., Martin-Gayo E., Leng J., Henrich T.J., Li J.Z. (2014). HIV-1 persistence in CD4^+^ T cells with stem cell-like properties. Nat. Med..

[65] Horiike M., Iwami S., Kodama M., Sato A., Watanabe Y., Yasui M., Ishida Y., Kobayashi T., Miura T., Igarashi T. (2012). Lymph nodes harbor viral reservoirs that cause rebound of plasma viremia in SIV-infected macaques upon cessation of combined antiretroviral therapy. Virology.

[66] Finzi D., Hermankova M., Pierson T., Carruth L.M., Buck C., Chaisson R.E., Quinn T.C., Chadwick K., Margolick J., Brookmeyer R. (1997). Identification of a reservoir for HIV-1 in patients on highly active antiretroviral therapy. Science.

[67] Koppensteiner H., Brack-Werner R., Schindler M. (2012). Macrophages and their relevance in Human Immunodeficiency Virus Type I infection. Retrovirology.

[68] Watters S.A., Mlcochova P., Gupta R.K. (2013). Macrophages: The neglected barrier to eradication. Curr. Opin. Infect. Dis..

[69] Spiegel H., Herbst H., Niedobitek G., Foss H.D., Stein H. (1992). Follicular dendritic cells are a major reservoir for human immunodeficiency virus type 1 in lymphoid tissues facilitating infection of CD4^+^ T-helper cells. Am. J. Pathol..

[70] Wang X., Ziani W., Xu H. (2016). Changes in Follicular CD4^+^ T Helper Cells as a Marker for Evaluating Disease Progression in the Competition between HIV and Host Immunity. Front. Immunol..

[71] Deenick E.K., Chan A., Ma C.S., Gatto D., Schwartzberg P.L., Brink R., Tangye S.G. (2010). Follicular helper T cell differentiation requires continuous antigen presentation that is independent of unique B cell signaling. Immunity.

[72] Baumjohann D., Preite S., Reboldi A., Ronchi F., Ansel K.M., Lanzavecchia A., Sallusto F. (2013). Persistent antigen and germinal center B cells sustain T follicular helper cell responses and phenotype. Immunity.

[73] Barnett L.G., Simkins H.M., Barnett B.E., Korn L.L., Johnson A.L., Wherry E.J., Wu G.F., Laufer T.M. (2014). B cell antigen presentation in the initiation of follicular helper T cell and germinal center differentiation. J. Immunol..

[74] Stieh D.J., Matias E., Xu H., Fought A.J., Blanchard J.L., Marx P.A., Veazey R.S., Hope T.J. (2016). Th17 Cells Are Preferentially Infected Very Early after Vaginal Transmission of SIV in Macaques. Cell Host Microbe.

[75] Ganor Y., Real F., Sennepin A., Dutertre C.A., Prevedel L., Xu L., Tudor D., Charmeteau B., Couedel-Courteille A., Marion S. (2019). HIV-1 reservoirs in urethral macrophages of patients under suppressive antiretroviral therapy. Nat. Microbiol..

[76] Abreu C.M., Veenhuis R.T., Avalos C.R., Graham S., Parrilla D.R., Ferreira E.A., Queen S.E., Shirk E.N., Bullock B.T., Li M. (2019). Myeloid and CD4 T Cells Comprise the Latent Reservoir in Antiretroviral Therapy-Suppressed SIVmac251-Infected Macaques. mBio.

[77] Wong M.E., Jaworowski A., Hearps A.C. (2019). The HIV Reservoir in Monocytes and Macrophages. Front. Immunol..

[78] DiNapoli S.R., Ortiz A.M., Wu F., Matsuda K., Twigg H.L., Hirsch V.M., Knox K., Brenchley J.M. (2017). Tissue-resident macrophages can contain replication-competent virus in antiretroviral-naive, SIV-infected Asian macaques. JCI Insight.

[79] Avalos C.R., Price S.L., Forsyth E.R., Pin J.N., Shirk E.N., Bullock B.T., Queen S.E., Li M., Gellerup D., O’Connor S.L. (2016). Quantitation of Productively Infected Monocytes and Macrophages of Simian Immunodeficiency Virus-Infected Macaques. J. Virol..

[80] Cattin A., Wiche Salinas T.R., Gosselin A., Planas D., Shacklett B., Cohen E.A., Ghali M.P., Routy J.P., Ancuta P. (2019). HIV-1 is rarely detected in blood and colon myeloid cells during viral-suppressive antiretroviral therapy. AIDS.

[81] Massanella M., Bakeman W., Sithinamsuwan P., Fletcher J.L.K., Chomchey N., Tipsuk S., Chalermchai T., Routy J.P., Ananworanich J., Valcour V.G. (2019). Infrequent HIV Infection of Circulating Monocytes during Antiretroviral Therapy. J. Virol..

[82] Abreu C.M., Veenhuis R.T., Avalos C.R., Graham S., Queen S.E., Shirk E.N., Bullock B.T., Li M., Metcalf Pate K.A., Beck S.E. (2019). Infectious Virus Persists in CD4^+^ T Cells and Macrophages in Antiretroviral Therapy-Suppressed Simian Immunodeficiency Virus-Infected Macaques. J. Virol..

[83] Jambo K.C., Banda D.H., Kankwatira A.M., Sukumar N., Allain T.J., Heyderman R.S., Russell D.G., Mwandumba H.C. (2014). Small alveolar macrophages are infected preferentially by HIV and exhibit impaired phagocytic function. Mucosal Immunol..

[84] Damouche A., Lazure T., Avettand-Fenoel V., Huot N., Dejucq-Rainsford N., Satie A.P., Melard A., David L., Gommet C., Ghosn J. (2015). Adipose Tissue Is a Neglected Viral Reservoir and an Inflammatory Site during Chronic HIV and SIV Infection. PLoS Pathog..

[85] Zalar A., Figueroa M.I., Ruibal-Ares B., Bare P., Cahn P., de Bracco M.M., Belmonte L. (2010). Macrophage HIV-1 infection in duodenal tissue of patients on long term HAART. Antivir. Res..

[86] Bernard-Stoecklin S., Gommet C., Corneau A.B., Guenounou S., Torres C., Dejucq-Rainsford N., Cosma A., Dereuddre-Bosquet N., Le Grand R. (2013). Semen CD4^+^ T cells and macrophages are productively infected at all stages of SIV infection in macaques. PLoS Pathog..

[87] Cenker J.J., Stultz R.D., McDonald D. (2017). Brain Microglial Cells Are Highly Susceptible to HIV-1 Infection and Spread. AIDS Res. Hum. Retrovir..

[88] Andrade V.M., Mavian C., Babic D., Cordeiro T., Sharkey M., Barrios L., Brander C., Martinez-Picado J., Dalmau J., Llano A. (2020). A minor population of macrophage-tropic HIV-1 variants is identified in recrudescing viremia following analytic treatment interruption. Proc. Natl. Acad. Sci. USA.

[89] Bruel T., Schwartz O. (2018). Markers of the HIV-1 reservoir: Facts and controversies. Curr. Opin. HIV AIDS.

[90] Darcis G., Berkhout B., Pasternak A.O. (2019). The Quest for Cellular Markers of HIV Reservoirs: Any Color You Like. Front. Immunol..

[91] Hurst J., Hoffmann M., Pace M., Williams J.P., Thornhill J., Hamlyn E., Meyerowitz J., Willberg C., Koelsch K.K., Robinson N. (2015). Immunological biomarkers predict HIV-1 viral rebound after treatment interruption. Nat. Commun..

[92] Fenwick C., Joo V., Jacquier P., Noto A., Banga R., Perreau M., Pantaleo G. (2019). T-cell exhaustion in HIV infection. Immunol. Rev..

[93] Iglesias-Ussel M., Vandergeeten C., Marchionni L., Chomont N., Romerio F. (2013). High levels of CD2 expression identify HIV-1 latently infected resting memory CD4^+^ T cells in virally suppressed subjects. J. Virol..

[94] Serra-Peinado C., Grau-Exposito J., Luque-Ballesteros L., Astorga-Gamaza A., Navarro J., Gallego-Rodriguez J., Martin M., Curran A., Burgos J., Ribera E. (2019). Expression of CD20 after viral reactivation renders HIV-reservoir cells susceptible to Rituximab. Nat. Commun..

[95] Biswas P., Mantelli B., Delfanti F., Ferrarini M., Poli G., Lazzarin A. (2003). CD30 ligation differentially affects CXCR4-dependent HIV-1 replication and soluble CD30 secretion in non-Hodgkin cell lines and in gamma delta T lymphocytes. Eur. J. Immunol..

[96] Descours B., Petitjean G., Lopez-Zaragoza J.L., Bruel T., Raffel R., Psomas C., Reynes J., Lacabaratz C., Levy Y., Schwartz O. (2017). CD32a is a marker of a CD4 T-cell HIV reservoir harbouring replication-competent proviruses. Nature.

[97] Hogan L.E., Vasquez J., Hobbs K.S., Hanhauser E., Aguilar-Rodriguez B., Hussien R., Thanh C., Gibson E.A., Carvidi A.B., Smith L.C.B. (2018). Increased HIV-1 transcriptional activity and infectious burden in peripheral blood and gut-associated CD4+ T cells expressing CD30. PLoS Pathog..

[98] Garcia M., Navarrete-Munoz M.A., Ligos J.M., Cabello A., Restrepo C., Lopez-Bernaldo J.C., de la Hera F.J., Barros C., Montoya M., Fernandez-Guerrero M. (2018). CD32 Expression is not Associated to HIV-DNA content in CD4 cell subsets of individuals with Different Levels of HIV Control. Sci. Rep..

[99] Abdel-Mohsen M., Kuri-Cervantes L., Grau-Exposito J., Spivak A.M., Nell R.A., Tomescu C., Vadrevu S.K., Giron L.B., Serra-Peinado C., Genesca M. (2018). CD32 is expressed on cells with transcriptionally active HIV but does not enrich for HIV DNA in resting T cells. Sci. Transl. Med..

[100] Osuna C.E., Lim S.Y., Kublin J.L., Apps R., Chen E., Mota T.M., Huang S.H., Ren Y., Bachtel N.D., Tsibris A.M. (2018). Evidence that CD32a does not mark the HIV-1 latent reservoir. Nature.

[101] Goncalves J., Moreira E., Sequeira I.J., Rodrigues A.S., Rueff J., Bras A. (2016). Integration of HIV in the Human Genome: Which Sites Are Preferential? A Genetic and Statistical Assessment. Int. J. Genom..

[102] Lee S.Y., Choi B.S., Yoon C.H., Kang C., Kim K., Kim K.C. (2019). Selection of biomarkers for HIV-1 latency by integrated analysis. Genomics.

[103] Calin R., Hamimi C., Lambert-Niclot S., Carcelain G., Bellet J., Assoumou L., Tubiana R., Calvez V., Dudoit Y., Costagliola D. (2016). Treatment interruption in chronically HIV-infected patients with an ultralow HIV reservoir. AIDS.

[104] Ananworanich J., Chomont N., Eller L.A., Kroon E., Tovanabutra S., Bose M., Nau M., Fletcher J.L.K., Tipsuk S., Vandergeeten C. (2016). HIV DNA Set Point is Rapidly Established in Acute HIV Infection and Dramatically Reduced by Early ART. EBioMedicine.

[105] Henrich T.J., Hatano H., Bacon O., Hogan L.E., Rutishauser R., Hill A., Kearney M.F., Anderson E.M., Buchbinder S.P., Cohen S.E. (2017). HIV-1 persistence following extremely early initiation of antiretroviral therapy (ART) during acute HIV-1 infection: An observational study. PLoS Med..

[106] Colby D.J., Trautmann L., Pinyakorn S., Leyre L., Pagliuzza A., Kroon E., Rolland M., Takata H., Buranapraditkun S., Intasan J. (2018). Rapid HIV RNA rebound after antiretroviral treatment interruption in persons durably suppressed in Fiebig I acute HIV infection. Nat. Med..

[107] Prendergast A., Mphatswe W., Tudor-Williams G., Rakgotho M., Pillay V., Thobakgale C., McCarthy N., Morris L., Walker B.D., Goulder P. (2008). Early virological suppression with three-class antiretroviral therapy in HIV-infected African infants. AIDS.

[108] Persaud D., Gay H., Ziemniak C., Chen Y.H., Piatak M., Chun T.W., Strain M., Richman D., Luzuriaga K. (2013). Absence of detectable HIV-1 viremia after treatment cessation in an infant. N. Engl. J. Med..

[109] Klein N., Palma P., Luzuriaga K., Pahwa S., Nastouli E., Gibb D.M., Rojo P., Borkowsky W., Bernardi S., Zangari P. (2015). Early antiretroviral therapy in children perinatally infected with HIV: A unique opportunity to implement immunotherapeutic approaches to prolong viral remission. Lancet Infect. Dis..

[110] Rainwater-Lovett K., Luzuriaga K., Persaud D. (2015). Very early combination antiretroviral therapy in infants: Prospects for cure. Curr. Opin. HIV AIDS.

[111] Pankau M.D., Wamalwa D., Benki-Nugent S., Tapia K., Ngugi E., Langat A., Otieno V., Moraa H., Maleche-Obimbo E., Overbaugh J. (2018). Decay of HIV DNA in the Reservoir and the Impact of Short Treatment Interruption in Kenyan Infants. Open Forum Infect. Dis..

[112] Katusiime M.G., Halvas E.K., Wright I., Joseph K., Bale M.J., Kirby-McCullough B., Engelbrecht S., Shao W., Hu W.S., Cotton M.F. (2020). Intact HIV Proviruses Persist in Children Seven to Nine Years after Initiation of Antiretroviral Therapy in the First Year of Life. J. Virol..

[113] Palma P., McManus M., Cotugno N., Rocca S., Rossi P., Luzuriaga K. (2020). The HIV-1 antibody response: A footprint of the viral reservoir in children vertically infected with HIV. Lancet HIV.

[114] Hutter G., Nowak D., Mossner M., Ganepola S., Mussig A., Allers K., Schneider T., Hofmann J., Kucherer C., Blau O. (2009). Long-term control of HIV by CCR5 Delta32/Delta32 stem-cell transplantation. N. Engl. J. Med..

[115] Gupta R.K., Abdul-Jawad S., McCoy L.E., Mok H.P., Peppa D., Salgado M., Martinez-Picado J., Nijhuis M., Wensing A.M.J., Lee H. (2019). HIV-1 remission following CCR5Delta32/Delta32 haematopoietic stem-cell transplantation. Nature.

[116] Scarborough R.J., Goguen R.P., Gatignol A. (2019). A second patient cured of HIV infection: Hopes and limitations. Virologie.

[117] Gupta R.K., Peppa D., Hill A.L., Galvez C., Salgado M., Pace M., McCoy L.E., Griffith S.A., Thornhill J., Alrubayyi A. (2020). Evidence for HIV-1 cure after CCR5Delta32/Delta32 allogeneic haemopoietic stem-cell transplantation 30 months post analytical treatment interruption: A case report. Lancet HIV.

[118] Whitney J.B., Hill A.L., Sanisetty S., Penaloza-MacMaster P., Liu J., Shetty M., Parenteau L., Cabral C., Shields J., Blackmore S. (2014). Rapid seeding of the viral reservoir prior to SIV viraemia in rhesus monkeys. Nature.

[119] Okoye A.A., Hansen S.G., Vaidya M., Fukazawa Y., Park H., Duell D.M., Lum R., Hughes C.M., Ventura A.B., Ainslie E. (2018). Early antiretroviral therapy limits SIV reservoir establishment to delay or prevent post-treatment viral rebound. Nat. Med..

[120] Pinkevych M., Fennessey C.M., Cromer D., Reid C., Trubey C.M., Lifson J.D., Keele B.F., Davenport M.P. (2019). Predictors of SIV recrudescence following antiretroviral treatment interruption. eLife.

[121] Wang X., Rasmussen T., Pahar B., Poonia B., Alvarez X., Lackner A.A., Veazey R.S. (2007). Massive infection and loss of CD4^+^ T cells occurs in the intestinal tract of neonatal rhesus macaques in acute SIV infection. Blood.

[122] Wang X., Xu H., Pahar B., Alvarez X., Green L.C., Dufour J., Moroney-Rasmussen T., Lackner A.A., Veazey R.S. (2010). Simian immunodeficiency virus selectively infects proliferating CD4^+^ T cells in neonatal rhesus macaques. Blood.

[123] Muenchhoff M., Prendergast A.J., Goulder P.J. (2014). Immunity to HIV in Early Life. Front. Immunol..

[124] Goulder P.J., Lewin S.R., Leitman E.M. (2016). Paediatric HIV infection: The potential for cure. Nat. Rev. Immunol..

[125] Violari A., Cotton M.F., Gibb D.M., Babiker A.G., Steyn J., Madhi S.A., Jean-Philippe P., McIntyre J.A., CHER Study Team (2008). Early antiretroviral therapy and mortality among HIV-infected infants. N. Engl. J. Med..

[126] Cotton M.F., Violari A., Otwombe K., Panchia R., Dobbels E., Rabie H., Josipovic D., Liberty A., Lazarus E., Innes S. (2013). Early time-limited antiretroviral therapy versus deferred therapy in South African infants infected with HIV: Results from the children with HIV early antiretroviral (CHER) randomised trial. Lancet.

[127] Luzuriaga K., Tabak B., Garber M., Chen Y.H., Ziemniak C., McManus M.M., Murray D., Strain M.C., Richman D.D., Chun T.W. (2014). HIV type 1 (HIV-1) proviral reservoirs decay continuously under sustained virologic control in HIV-1-infected children who received early treatment. J. Infect. Dis..

[128] Van Zyl G.U., Bedison M.A., van Rensburg A.J., Laughton B., Cotton M.F., Mellors J.W. (2015). Early Antiretroviral Therapy in South African Children Reduces HIV-1-Infected Cells and Cell-Associated HIV-1 RNA in Blood Mononuclear Cells. J. Infect. Dis..

[129] Martinez-Bonet M., Puertas M.C., Fortuny C., Ouchi D., Mellado M.J., Rojo P., Noguera-Julian A., Munoz-Fernandez M.A., Martinez-Picado J. (2015). Establishment and Replenishment of the Viral Reservoir in Perinatally HIV-1-infected Children Initiating Very Early Antiretroviral Therapy. Clin. Infect. Dis..

[130] Garcia-Broncano P., Maddali S., Einkauf K.B., Jiang C., Gao C., Chevalier J., Chowdhury F.Z., Maswabi K., Ajibola G., Moyo S. (2019). Early antiretroviral therapy in neonates with HIV-1 infection restricts viral reservoir size and induces a distinct innate immune profile. Sci. Transl. Med..

[131] Giacomet V., Trabattoni D., Zanchetta N., Biasin M., Gismondo M., Clerici M., Zuccotti G. (2014). No cure of HIV infection in a child despite early treatment and apparent viral clearance. Lancet.

[132] Luzuriaga K., Gay H., Ziemniak C., Sanborn K.B., Somasundaran M., Rainwater-Lovett K., Mellors J.W., Rosenbloom D., Persaud D. (2015). Viremic relapse after HIV-1 remission in a perinatally infected child. N. Engl. J. Med..

[133] Butler K.M., Gavin P., Coughlan S., Rochford A., Mc Donagh S., Cunningham O., Poulsom H., Watters S.A., Klein N. (2015). Rapid viral rebound after 4 years of suppressive therapy in a seronegative HIV-1 infected infant treated from birth. Pediatr. Infect. Dis. J..

[134] Ateba Ndongo F., Texier G., Ida Penda C., Tejiokem M.C., Tetang Ndiang S., Ndongo J.A., Guemkam G., Sofeu C.L., Kfutwah A., Faye A. (2018). Virologic Response to Early Antiretroviral Therapy in HIV-infected Infants: Evaluation After 2 Years of Treatment in the Pediacam Study, Cameroon. Pediatr. Infect. Dis. J..

[135] Kuhn L., Strehlau R., Shiau S., Patel F., Shen Y., Technau K.G., Burke M., Sherman G., Coovadia A., Aldrovandi G.M. (2020). Early antiretroviral treatment of infants to attain HIV remission. EClinicalMedicine.

[136] Luzuriaga K. (2016). Early Combination Antiretroviral Therapy Limits HIV-1 Persistence in Children. Annu. Rev. Med..

[137] Simonetti F.R., White J.A., Tumiotto C., Ritter K.D., Cai M., Gandhi R.T., Deeks S.G., Howell B.J., Montaner L.J., Blankson J.N. (2020). Intact proviral DNA assay analysis of large cohorts of people with HIV provides a benchmark for the frequency and composition of persistent proviral DNA. Proc. Natl. Acad. Sci. USA.

[138] Clarridge K.E., Blazkova J., Einkauf K., Petrone M., Refsland E.W., Justement J.S., Shi V., Huiting E.D., Seamon C.A., Lee G.Q. (2018). Effect of analytical treatment interruption and reinitiation of antiretroviral therapy on HIV reservoirs and immunologic parameters in infected individuals. PLoS Pathog..

[139] Abdel-Mohsen M., Richman D., Siliciano R.F., Nussenzweig M.C., Howell B.J., Martinez-Picado J., Chomont N., Bar K.J., Yu X.G., Lichterfeld M. (2020). Recommendations for measuring HIV reservoir size in cure-directed clinical trials. Nat. Med..

[140] Hodel F., Patxot M., Snaka T., Ciuffi A. (2016). HIV-1 latent reservoir: Size matters. Future Virol..

[141] Mexas A.M., Graf E.H., Pace M.J., Yu J.J., Papasavvas E., Azzoni L., Busch M.P., Di Mascio M., Foulkes A.S., Migueles S.A. (2012). Concurrent measures of total and integrated HIV DNA monitor reservoirs and ongoing replication in eradication trials. AIDS.

[142] Yukl S.A., Kaiser P., Kim P., Telwatte S., Joshi S.K., Vu M., Lampiris H., Wong J.K. (2018). HIV latency in isolated patient CD4^+^ T cells may be due to blocks in HIV transcriptional elongation, completion, and splicing. Sci. Transl. Med..

[143] Rutsaert S., Bosman K., Trypsteen W., Nijhuis M., Vandekerckhove L. (2018). Digital PCR as a tool to measure HIV persistence. Retrovirology.

[144] Ho Y.C., Shan L., Hosmane N.N., Wang J., Laskey S.B., Rosenbloom D.I., Lai J., Blankson J.N., Siliciano J.D., Siliciano R.F. (2013). Replication-competent noninduced proviruses in the latent reservoir increase barrier to HIV-1 cure. Cell.

[145] Imamichi H., Dewar R.L., Adelsberger J.W., Rehm C.A., O’Doherty U., Paxinos E.E., Fauci A.S., Lane H.C. (2016). Defective HIV-1 proviruses produce novel protein-coding RNA species in HIV-infected patients on combination antiretroviral therapy. Proc. Natl. Acad. Sci. USA.

[146] Bruner K.M., Murray A.J., Pollack R.A., Soliman M.G., Laskey S.B., Capoferri A.A., Lai J., Strain M.C., Lada S.M., Hoh R. (2016). Defective proviruses rapidly accumulate during acute HIV-1 infection. Nat. Med..

[147] Siliciano J.D., Siliciano R.F. (2018). Assays to Measure Latency, Reservoirs, and Reactivation. Curr. Top. Microbiol. Immunol..

[148] Massanella M., Richman D.D. (2016). Measuring the latent reservoir in vivo. J. Clin. Investig..

[149] Plantin J., Massanella M., Chomont N. (2018). Inducible HIV RNA transcription assays to measure HIV persistence: Pros and cons of a compromise. Retrovirology.

[150] Falcinelli S.D., Ceriani C., Margolis D.M., Archin N.M. (2019). New Frontiers in Measuring and Characterizing the HIV Reservoir. Front. Microbiol..

[151] Battivelli E., Dahabieh M.S., Abdel-Mohsen M., Svensson J.P., Tojal Da Silva I., Cohn L.B., Gramatica A., Deeks S., Greene W.C., Pillai S.K. (2018). Distinct chromatin functional states correlate with HIV latency reactivation in infected primary CD4^+^ T cells. eLife.

[152] Kwon K.J., Timmons A.E., Sengupta S., Simonetti F.R., Zhang H., Hoh R., Deeks S.G., Siliciano J.D., Siliciano R.F. (2020). Different human resting memory CD4^+^ T cell subsets show similar low inducibility of latent HIV-1 proviruses. Sci. Transl. Med..

[153] Jiang C., Lian X., Gao C., Sun X., Einkauf K.B., Chevalier J.M., Chen S.M.Y., Hua S., Rhee B., Chang K. (2020). Distinct viral reservoirs in individuals with spontaneous control of HIV-1. Nature.

[154] Hiener B., Horsburgh B.A., Eden J.S., Barton K., Schlub T.E., Lee E., von Stockenstrom S., Odevall L., Milush J.M., Liegler T. (2017). Identification of Genetically Intact HIV-1 Proviruses in Specific CD4^+^ T Cells from Effectively Treated Participants. Cell Rep..

[155] Vandergeeten C., Fromentin R., DaFonseca S., Lawani M.B., Sereti I., Lederman M.M., Ramgopal M., Routy J.P., Sekaly R.P., Chomont N. (2013). Interleukin-7 promotes HIV persistence during antiretroviral therapy. Blood.

[156] Hughes S.H., Coffin J.M. (2016). What Integration Sites Tell Us about HIV Persistence. Cell Host Microbe.

[157] Antar A.A.R., Jenike K.M., Jang S., Rigau D.N., Reeves D.B., Hoh R., Krone M.R., Keruly J.C., Moore R.D., Schiffer J.T. (2020). Longitudinal study reveals HIV-1-infected CD4^+^ T cell dynamics during long-term antiretroviral therapy. J. Clin. Investig..

[158] Pollack R.A., Jones R.B., Pertea M., Bruner K.M., Martin A.R., Thomas A.S., Capoferri A.A., Beg S.A., Huang S.H., Karandish S. (2017). Defective HIV-1 Proviruses Are Expressed and Can Be Recognized by Cytotoxic T Lymphocytes, which Shape the Proviral Landscape. Cell Host Microbe.

[159] Sharaf R., Lee G.Q., Sun X., Etemad B., Aboukhater L.M., Hu Z., Brumme Z.L., Aga E., Bosch R.J., Wen Y. (2018). HIV-1 proviral landscapes distinguish posttreatment controllers from noncontrollers. J. Clin. Investig..

[160] Einkauf K.B., Lee G.Q., Gao C., Sharaf R., Sun X., Hua S., Chen S.M., Jiang C., Lian X., Chowdhury F.Z. (2019). Intact HIV-1 proviruses accumulate at distinct chromosomal positions during prolonged antiretroviral therapy. J. Clin. Investig..

[161] Bui J.K., Sobolewski M.D., Keele B.F., Spindler J., Musick A., Wiegand A., Luke B.T., Shao W., Hughes S.H., Coffin J.M. (2017). Proviruses with identical sequences comprise a large fraction of the replication-competent HIV reservoir. PLoS Pathog..

[162] Wiegand A., Spindler J., Hong F.F., Shao W., Cyktor J.C., Cillo A.R., Halvas E.K., Coffin J.M., Mellors J.W., Kearney M.F. (2017). Single-cell analysis of HIV-1 transcriptional activity reveals expression of proviruses in expanded clones during ART. Proc. Natl. Acad. Sci. USA.

[163] Baxter A.E., O’Doherty U., Kaufmann D.E. (2018). Beyond the replication-competent HIV reservoir: Transcription and translation-competent reservoirs. Retrovirology.

[164] Wang Z., Simonetti F.R., Siliciano R.F., Laird G.M. (2018). Measuring replication competent HIV-1: Advances and challenges in defining the latent reservoir. Retrovirology.

[165] Blankson J.N., Persaud D., Siliciano R.F. (2002). The challenge of viral reservoirs in HIV-1 infection. Annu. Rev. Med..

[166] Elsheikh M.M., Tang Y., Li D., Jiang G. (2019). Deep latency: A new insight into a functional HIV cure. EBioMedicine.

[167] Sgadari C., Monini P., Tripiciano A., Picconi O., Casabianca A., Orlandi C., Moretti S., Francavilla V., Arancio A., Paniccia G. (2019). Continued Decay of HIV Proviral DNA Upon Vaccination With HIV-1 Tat of Subjects on Long-Term ART: An 8-Year Follow-Up Study. Front. Immunol..

[168] Pitisuttithum P., Nitayaphan S., Chariyalertsak S., Kaewkungwal J., Dawson P., Dhitavat J., Phonrat B., Akapirat S., Karasavvas N., Wieczorek L. (2020). Late boosting of the RV144 regimen with AIDSVAX B/E and ALVAC-HIV in HIV-uninfected Thai volunteers: A double-blind, randomised controlled trial. Lancet HIV.

[169] Spivak A.M., Planelles V. (2018). Novel Latency Reversal Agents for HIV-1 Cure. Annu. Rev. Med..

[170] Bashiri K., Rezaei N., Nasi M., Cossarizza A. (2018). The role of latency reversal agents in the cure of HIV: A review of current data. Immunol. Lett..

[171] Thomas J., Ruggiero A., Paxton W.A., Pollakis G. (2020). Measuring the Success of HIV-1 Cure Strategies. Front. Cell. Infect. Microbiol..

[172] Darcis G., Van Driessche B., Van Lint C. (2017). HIV Latency: Should We Shock or Lock?. Trends Immunol..

[173] Caskey M. (2020). Broadly neutralizing antibodies for the treatment and prevention of HIV infection. Curr. Opin. HIV AIDS.

[174] Jacobson J.M., Khalili K. (2018). Toward the Cure of HIV-1 Infection: Lessons Learned and Yet to be Learned as New Strategies are Developed. AIDS Rev..

[175] Atkins A.J., Allen A.G., Dampier W., Haddad E.K., Nonnemacher M.R., Wigdahl B. (2020). HIV-1 cure strategies: Why CRISPR?. Expert Opin. Biol. Ther..

[176] Deeks S.G. (2012). HIV: Shock and kill. Nature.

[177] Archin N.M., Margolis D.M. (2014). Emerging strategies to deplete the HIV reservoir. Curr. Opin. Infect. Dis..

[178] Cillo A.R., Sobolewski M.D., Bosch R.J., Fyne E., Piatak M., Coffin J.M., Mellors J.W. (2014). Quantification of HIV-1 latency reversal in resting CD4+ T cells from patients on suppressive antiretroviral therapy. Proc. Natl. Acad. Sci. USA.

[179] Del Prete G.Q., Shoemaker R., Oswald K., Lara A., Trubey C.M., Fast R., Schneider D.K., Kiser R., Coalter V., Wiles A. (2014). Effect of SAHA administration on the residual virus pool in a model of combination antiretroviral therapy-mediated suppression in SIVmac239-infected Indian rhesus macaques. Antimicrob. Agents Chemother..

[180] Mohammadi P., di Iulio J., Munoz M., Martinez R., Bartha I., Cavassini M., Thorball C., Fellay J., Beerenwinkel N., Ciuffi A. (2014). Dynamics of HIV latency and reactivation in a primary CD4^+^ T cell model. PLoS Pathog..

[181] Lucera M.B., Tilton C.A., Mao H., Dobrowolski C., Tabler C.O., Haqqani A.A., Karn J., Tilton J.C. (2014). The histone deacetylase inhibitor vorinostat (SAHA) increases the susceptibility of uninfected CD4^+^ T cells to HIV by increasing the kinetics and efficiency of postentry viral events. J. Virol..

[182] Jones R.B., O’Connor R., Mueller S., Foley M., Szeto G.L., Karel D., Lichterfeld M., Kovacs C., Ostrowski M.A., Trocha A. (2014). Histone deacetylase inhibitors impair the elimination of HIV-infected cells by cytotoxic T-lymphocytes. PLoS Pathog..

[183] Richman D.D., Margolis D.M., Delaney M., Greene W.C., Hazuda D., Pomerantz R.J. (2009). The challenge of finding a cure for HIV infection. Science.

[184] Marsden M.D., Zack J.A. (2009). Eradication of HIV: Current challenges and new directions. J. Antimicrob. Chemother..

[185] Kim Y., Anderson J.L., Lewin S.R. (2018). Getting the “Kill” into “Shock and Kill”: Strategies to Eliminate Latent HIV. Cell Host Microbe.

[186] Niu Q., Liu Z., Alamer E., Fan X., Chen H., Endsley J., Gelman B.B., Tian B., Kim J.H., Michael N.L. (2019). Structure-guided drug design identifies a BRD4-selective small molecule that suppresses HIV. J. Clin. Investig..

[187] Pincus S.H., Song K., Maresh G.A., Frank A., Worthylake D., Chung H.K., Polacino P., Hamer D.H., Coyne C.P., Rosenblum M.G. (2017). Design and In Vivo Characterization of Immunoconjugates Targeting HIV gp160. J. Virol..

[188] Berger E.A. (2011). Targeted cytotoxic therapy: Adapting a rapidly progressing anticancer paradigm for depletion of persistent HIV-infected cell reservoirs. Curr. Opin. HIV AIDS.

[189] Niessl J., Baxter A.E., Mendoza P., Jankovic M., Cohen Y.Z., Butler A.L., Lu C.L., Dube M., Shimeliovich I., Gruell H. (2020). Combination anti-HIV-1 antibody therapy is associated with increased virus-specific T cell immunity. Nat. Med..

[190] Liu Y., Cao W., Sun M., Li T. (2020). Broadly neutralizing antibodies for HIV-1: Efficacies, challenges and opportunities. Emerg. Microbes Infect..

[191] Cohen Y.Z., Caskey M. (2018). Broadly neutralizing antibodies for treatment and prevention of HIV-1 infection. Curr. Opin. HIV AIDS.

[192] Haynes B.F., Burton D.R., Mascola J.R. (2019). Multiple roles for HIV broadly neutralizing antibodies. Sci. Transl. Med..

[193] Sok D., Burton D.R. (2018). Recent progress in broadly neutralizing antibodies to HIV. Nat. Immunol..

[194] Jaworski J.P., Cahn P. (2018). Preventive and therapeutic features of broadly neutralising monoclonal antibodies against HIV-1. Lancet HIV.

[195] Tuyishime M., Garrido C., Jha S., Moeser M., Mielke D., LaBranche C., Montefiori D., Haynes B.F., Joseph S., Margolis D.M. (2020). Improved killing of HIV-infected cells using three neutralizing and non-neutralizing antibodies. J. Clin. Investig..

[196] Panfil A.R., London J.A., Green P.L., Yoder K.E. (2018). CRISPR/Cas9 Genome Editing to Disable the Latent HIV-1 Provirus. Front. Microbiol..

[197] Xiao Q., Guo D., Chen S. (2019). Application of CRISPR/Cas9-Based Gene Editing in HIV-1/AIDS Therapy. Front. Cell. Infect. Microbiol..

[198] Das A.T., Binda C.S., Berkhout B. (2019). Elimination of infectious HIV DNA by CRISPR-Cas9. Curr. Opin. Virol..

